# Metamemory and Memory Discrepancies in Directed Forgetting of Emotional Information

**DOI:** 10.5964/ejop.2567

**Published:** 2021-02-26

**Authors:** Dicle Çapan, Simay Ikier

**Affiliations:** a Department of Psychology, Koç University, Istanbul, Turkey; b Department of Psychology, Bahçeşehir University, Istanbul, Turkey; University of South Wales, Pontypridd, United Kingdom

**Keywords:** memory, directed forgetting, metamemory, judgment of learning, emotion

## Abstract

Directed Forgetting (DF) studies show that it is possible to exert cognitive control to intentionally forget information. The aim of the present study was to investigate how aware individuals are of the control they have over what they remember and forget when the information is emotional. Participants were presented with positive, negative and neutral photographs, and each photograph was followed by either a Remember or a Forget instruction. Then, for each photograph, participants provided Judgments of Learning (JOLs) by indicating their likelihood of recognizing that item on a subsequent test. In the recognition phase, participants were asked to indicate all old items, irrespective of instruction. Remember items had higher JOLs than Forget items for all item types, indicating that participants believe they can intentionally forget even emotional information—which is not the case based on the actual recognition results. DF effect, which was calculated by subtracting recognition for Forget items from Remember ones was only significant for neutral items. Emotional information disrupted cognitive control, eliminating the DF effect. Response times for JOLs showed that evaluation of emotional information, especially negatively emotional information takes longer, and thus is more difficult. For both Remember and Forget items, JOLs reflected sensitivity to emotionality of the items, with emotional items receiving higher JOLs than the neutral ones. Actual recognition confirmed better recognition for only negative items but not for positive ones. JOLs also reflected underestimation of actual recognition performance. Discrepancies in metacognitive judgments due to emotional valence as well as the reasons for underestimation are discussed.

While individuals can form hypotheses and inferences about their outer environment, they can also do these about their own mental processes (Koriat, 1997; Undorf, Söllner, & Bröder, 2018). They can monitor, regulate, and control their mental processes, and they can provide predictions about their mental abilities in the form of metacognitive judgments (Koriat & Helstrup, 2007; Undorf et al., 2018). One type of judgment that they can provide is Judgment of Learning (JOL) which is a prediction about the future likelihood of retrieval for recently learned information (Hourihan & Bursey, 2017; for a review, see Rhodes, 2016). Recent research showed that emotional valence of the information to be remembered can affect JOLs, with higher JOLs given to emotional than to neutral information (Undorf et al., 2018). Emotional intensity also increases JOLs (Hourihan, Fraundorf, & Benjamin, 2017). These findings indicate that individuals believe emotional aspects of the information will increase their likelihood of remembering that information. In fact, this judgment is found to be correct in studies using emotional materials. Memory is enhanced for both of positive and negative information (e.g., Hess, Popham, Dennis, & Emery, 2013) or at least for negative information (e.g., Humphreys, Underwood, & Chapman, 2010).

While metacognitive judgments are sometimes accurate predictions of memory performance (e.g., Zimmerman & Kelley, 2010), there are also findings showing discrepancies between JOLs and actual memory (for a review, see Bjork, Dunlosky, & Kornell, 2013). Nomi, Rhodes, and Clearly (2013) found that face photographs with negative and positive emotional expressions received higher JOLs than the ones with neutral expressions. This was not reflected in actual recognition, in which neutral items were remembered better.

While JOLs may be provided on the basis of cues such as repetitions, instructions, and the context of encoding for the information and reflect conscious, theory-based reasoning (Hourihan et al., 2017), they may also be based on other cues such as subjective feelings about how well a stimulus has been learned. These internal cues are less consciously detectible (e.g., Serra & Metcalfe, 2009). Emotionality of the information may become a salient cue and the content may arise a feeling of fluency (Koriat, 1997). In the study by Hourihan and Bursey (2017), JOLs for positive and neutral photographs were compared and the results showed that while positive photographs were given higher JOLs than the neutral photographs, there was no benefit of emotional content on actual recognition. They explained this result by arguing that emotional and neutral content arise different physiological responses, and positive feelings experienced due to viewing positive photographs result in misjudgments of higher recognition. Thus, especially in the case of emotional items, reliance on feelings rather than conscious reasoning in judgments may decrease accuracy.

While previous studies focused on beliefs about how well emotional information can be remembered, to our knowledge, there is no prior study investigating beliefs about how well emotional information can be forgotten. In fact, even studies investigating metacognitive judgments about forgetting neutral information are limited in number (Sahakyan & Foster, 2016). Forgetting is also an integral part of memory and despite its negative connotation; it is functional and desirable to some extent. Evidence indicates that individuals can actually forget information intentionally (Hauswald & Kissler, 2008). Intentional forgetting can increase the efficiency of memory, by preventing unnecessary information from interfering with currently relevant information (e.g., Hauswald & Kissler, 2008; Ullsperger, Mecklinger, & Müller, 2000). The aim of the present stud was to investigate individuals’ ability to predict their own intentional forgetting when the information is emotional.

Intentional forgetting is commonly studied by using a directed forgetting (DF) paradigm. In commonly used DF paradigms, participants are asked to remember some items that are presented and to forget the others. Memory for all items is tested at retrieval. These paradigms reveal a DF effect; with better memory for remember than forget items (e.g., Basden, 1996; Basden, Basden, & Gargano, 1993).

Previous emotional DF studies generally showed that negatively emotional information is more difficult to intentionally forget. Tay and Yang (2017) investigated DF of emotional faces and found that angry faces were more resistant to forgetting than positive ones. In a study by Payne and Corrigan (2007), pleasant, unpleasant and neutral pictures were used, and DF was observed for neutral pictures but not for emotional ones. Otani et al. (2012) showed DF of neutral and positive pictures, but not of negative ones. These findings supported the hypothesis that there is stronger memory for negative materials (Kensinger & Corkin, 2004).

While the fact that individuals can intentionally forget indicates that cognitive control can be exerted over the contents of the mind (Sahakyan & Foster, 2016), the studies outlined above show that the emotionality of the information could disrupt control. One question that remains is whether individuals are aware of their ability to intentionally forget and of the disruptions in this ability due to the emotionality of the items. Friedman and Castel (2011) investigated JOLs for DF by using emotionally neutral items. They presented participants with words and asked them to provide JOLs for each word, by indicating the likelihood that they would remember that word in a follow-up test, using a scale from 0 to 100%. The results showed that individuals gave higher recall prediction for remember items than forget ones, indicating a metacognitive awareness of their ability to follow instructions, and to control what they will remember and forget, despite a general overconfidence in their predictions.

The present study aimed to investigate JOLs for emotional information in a DF paradigm. Questions of interest were whether remember versus forget instructions and emotional valence affect JOLs. To this end, each photograph in the DF paradigm either had a positive, negative or neutral valence, and each item was followed by either a remember or a forget instruction. Higher JOLs were hypothesized for remember than forget items. Despite the absence of studies investigating JOLs for emotional items to-be-forgotten, based on the literature showing higher JOLs for emotional items to-be-remembered, it was hypothesized that also for to-be-forgotten information, emotional items would have higher JOLs than neutral ones. With regards to emotional DF, a reduced DF effect for emotional items was expected. JOLs were compared to actual recognition for remember versus forget items of different valence.

## The Pilot Study

### Method

#### Participants

A pilot study was conducted with 20 participants (10 females and 10 males), with an average age of *M* = 21.92, prior to the present study to select photographs. Participants in the pilot study were at least high school graduates.

#### Materials and Procedure

The study was approved by Yeditepe University’s Humanities and Social Research Ethics Review Board. Informed consent was obtained from all participants. For the pilot study, 237 photographs (87 positive, 77 negative and 73 neutral) were selected from the International Affective Picture System (IAPS; Lang, Bradley, & Cuthbert, 1999) by excluding the ones that are culture-specific (e.g., church) or extremely negatively emotional (e.g., dead bodies). Participants provided valence and arousal ratings for all photographs on 7-point scales with higher points corresponding to higher positivity and higher arousal. Participants were debriefed.

### Results

Alpha level was taken as .05. Bonferroni corrections were applied in the pairwise comparisons and Greenhouse-Geisser corrections were applied when Mauchly’s Test indicated that sphericity was violated.

For the present study, 180 target photographs (60 negative, 60 positive and 60 neutral) were selected. Two neutral items were also selected to serve as buffers. The selected photographs differed in valence, *F*(1,71) = 11917.22, *p* < .001, $\eta_{p}^{2}$ = .995, with higher positivity ratings for positive (*M* = 5.45, *SE* = 0.05) than negative (*M* = 2.04, *SE* = 0.95), *p* < .01, and neutral (*M* = 3.93, *SE* = 0.03), *p* < .01, and for neutral than negative (*M* = 2.04, *SE* = 0.05) photographs, *p* < .01. Photographs also differed in arousal, *F*(2, 107) = 233.462, *p* < .01, $\eta_{p}^{2}$ = .80. Specifically, negative (*M* = 5.05, *SE* = 0.04) and neutral photographs (*M* = 3.72, *SE* = 0.04), *p* < .01, and positive (*M* = 4.91, *SE* = 0.06) and neutral photographs, *p* < .01 had different arousal ratings. Negative and positive photographs did not differ in arousal, *p* = .16.

## The Present Study

### Method

#### Participants

Sixty participants (30 females and 30 males, *M_age_* = 22.62, *SE* = 0.28) who did not participate in the pilot study participated in the present study. Participants were at least high school graduates with an average education of *M* = 14.85 years, *SE* = 1.36. They reported no history of neurological or psychiatric disorders.

#### Materials and Procedure

The study was approved by Yeditepe University’s Humanities and Social Research Ethics Review Board. Informed consent was obtained from all participants. Participants were tested individually in the laboratory. First, they completed the Positive Negative Affect Schedule (PANAS; Watson, Clark, & Tellegen, 1988, standardized into Turkish by Gençöz, 2000) as a measure of their positive and negative mood. All visual stimuli (image size 650 × 650 pixels) were displayed using Visual Studio 2015 (C#) on a MacBook Pro with a 13-inch monitor that was about 50 cm away from the participant. In the study phase, participants were presented with 120 of the critical photographs (40 positive, 40 negative, 40 neutral). Critical photographs were each presented for 2,500 ms, in random order. Half of the photographs were followed by a Remember instruction, and the other half by a Forget instruction, by providing the initial letters of the words Remember and Forget, four times after each item, similar to other studies (e.g., Otani et al., 2012). Remember and Forget instructions were presented for 2,000 ms, in black Arial 48-point font, with uppercase letters on a white background. Photographs were counterbalanced across participants such that a photograph with a particular valence occurred as a Remember versus a Forget item an equal number of times across participants. Participants were asked to remember only the Remember photographs for a recognition test, and to try to forget the Forget photographs.

Following each Remember/Forget instruction, participants provided JOLs for each item. They indicated their likelihood of recognizing that photograph on a follow-up memory test as a percentage, on a scale ranging from 0 to 100%. Participants were told that 0% indicates that they definitely would not recognize the photograph, whereas 100% indicates that they would definitely recognize it, and that they can provide the values in between to indicate their likelihood of recognition accordingly.

Each JOL rating was followed by a blank screen that appeared for 1,500 ms, and then the next trial followed. Trials were divided into three blocks, consisting of 40 trials each. After each block, a seven second break was given in which participants could relax. There was a neutral buffer at the beginning of the first block, and another one at the end of the last block.

The study session was followed by a filled interval in which participants were engaged in a non-verbal task for approximately 3 minutes on the computer (i.e., Tower of Hanoi problem). Subsequently, the recognition phase for all items followed. In the recognition phase, all critical photographs from the study phase, plus 60 new photographs were presented for 1,500 ms each, in random order. Participants made a forced-choice old-new decision for each photograph, by pressing a button on the screen by using the mouse, as quickly as possible. All participants were debriefed.

### Results

For all statistical analyses, alpha level was taken as .05. In all repeated measures Analyses of Variance, Bonferroni corrections were applied in the pairwise comparisons and Greenhouse-Geisser corrections were applied when Mauchly’s Test indicated that sphericity was violated.

Means and response times for JOLs are presented in Table 1, separately for each item type. JOLs for remember items were higher than forget items, for all item types, for positive items, *t*(59) = 8.71, *p* < .001, *d* = 0.65, for negative items, *t*(59) = 5.65, *p* < .001, *d* = 0.54, and for neutral items, *t*(59) = 7.00, *p* < .001, *d* = 0.55. Response times (RTs) were longer for forget items than remember items, for negative, *t*(59) = 2.59, *p* < .05, *d* = 0.21, and for positive *t*(59) = 2.08, *p* < .05, *d* = 0.21 items, but not for neutral ones, *t*(59) = 1.80, *p* = .08.

**Table 1:** Mean JOL Ratings (%) and Mean RTs (ms) for JOLs

Item instruction	Item valence											
	Positive				Negative				Neutral			
	*M*	*SE*	RTs	*SE*	*M*	*SE*	RTs	*SE*	*M*	*SE*	RTs	*SE*
Remember	56.64	2.51	2.654	139	61.59	2.47	2.776	149	43.68	2.63	2.530	126
Forget	44.51	2.34	2.910	170	51.35	2.41	2.945	143	33.27	2.25	2.666	142

*Note.* *N* = 60. JOL = Judgments of Learning; RTs = Response Times.

Means for recognition of the old items and false alarms (FAs) for new items are presented in Table 2, separately for each item type. First, a DF effect was calculated by subtracting recognition for forget items from remember items. DF effect was 1.17 % for positive (*SE* = 1.11), 0.83% for negative (*SE* = 0.98) and 2.42 % for neutral (*SE* = 1.08) items. Single sample *t*-test showed that the effect was significant for only neutral items, *t*(59) = 2.24, *p* < .05, *d* = 0.29. The other effects were statistically equal to zero, *p*s > .30.

**Table 2:** Mean Recognition for Targets and False Alarms (FAs) for New Items (%)

Item type	Item valence					
	Positive		Negative		Neutral	
	*M*	*SE*	*M*	*SE*	*M*	*SE*
Remember	88.92	1.15	93.00	1.02	91.50	1.37
Forget	87.75	1.38	92.17	1.00	89.08	1.53
FAs	2.17	0.54	5.75	0.93	2.58	0.44

*Note.* *N* = 60.

JOLs for remember items were different for the three item types, *F*(2, 97) = 49.43, *p* < .001, $\eta_{p}^{2}$ = .46. Pairwise comparisons showed that JOLs for negative items were higher than positive, *p* < .01, and neutral *p* < .001 items. JOLs for positive items were also higher than neutral ones, *p* < .001. Higher JOLs for emotional items were not reflected in actual recognition of remember items. Although recognition of remember items differed for the three item types, *F*(2, 110) = 5.39, *p* < .01, $\eta_{p}^{2}$ = .08, pairwise comparisons showed that the difference was only significant between negative and positive items, with higher recognition of negative ones, *p* < .01. The other comparisons were non-significant, *p*s > .22. RTs for providing the JOLs were different for the item types, *F*(2, 118) = 4.79, *p* < .05, $\eta_{p}^{2}$ = .08. It took longer for participants to provide JOLs only for negative items than neutral ones, *p* < .05. The other comparisons were non-significant, *p*s > .24.

JOLs for forget items were also different for the three item types, *F*(2, 101) = 72.08, *p* < .001, $\eta_{p}^{2}$ = .55. JOLs for each item type differed from the JOLs for the other two item types, *p*s < .001, with highest JOLs for negative items, followed by positive ones. Actual recognition of forget items resembled the remember items: Despite the overall difference, *F*(2, 118) = 6.02, *p* < .01, $\eta_{p}^{2}$ = .09, pairwise comparisons showed a significant difference between only negative and positive items, *p* < .01. The other comparisons were non-significant, *p*s > .08. Overall RTs for JOLs for forget items were different for the three item types, *F*(2, 106) = 7.70, *p* < .001, $\eta_{p}^{2}$ = .11. Pairwise comparisons showed that emotionality of the items made participants respond slower, with significant differences between positive and neutral, *p* < .05, and negative and neutral, *p* < .001 items, but not between positive and negative ones, *p* > .05. Overall RTs for JOLs for forget items were different for the three item types, *F*(2, 58) = 9.82, *p* < .001. Pairwise comparisons showed that emotionality of the items made participants respond slower, with significant differences between positive and neutral, *p* < .05, and negative and neutral, *p* < .001 items, but not between positive and negative ones, *p* > .05.

For all item types, JOLs were significantly lower than actual recognition, all *p*s < .05.

It should be noted that FAs differed for item types as well, *F*(2, 97) = 13.90, *p* < .001, $\eta_{p}^{2}$ = .19. Specifically, FAs for new negative items were higher than both new positive, *p* < .001 and new neutral items, *p* < .01. FAs for positive and neutral items did not differ, *p* = 1.00. Thus, despite a recognition advantage, negative items also lead to higher FAs.

PANAS ratings revealed average positive affect *M* = 35.65, *SE* = 1.12, and rather low negative affect, *M* = 22.88, *SE* = 1.06. PANAS positive scores showed significant positive correlations with the recognition of positive Remember, *r* = .37, *N* = 60, *p* < .01 and positive Forget photographs, *r* = .29, *N* = 60, *p* < .05. JOL ratings or JOL RTs for positive items did not correlate with PANAS positive scores, all *p*s > .06. PANAS negative scores showed significant positive correlations with the recognition of negative Remember, *r* = −.42, *N* = 60, *p* < .01 and negative Forget photographs, *r* = −29, *N* = 60, *p* < .05. JOL ratings or JOL RTs for negative items did not correlate with PANAS negative scores, all *p*s > .18.

## Discussion

In the present study, higher JOLs were hypothesized for remember than forget items. This was confirmed by the results showing higher JOLs for positive, negative, and neutral remember items than corresponding forget ones. This finding is consistent with findings indicating sensitivity to DF instructions in JOLs (Friedman & Castel, 2011) and additionally shows that participants believe they can intentionally forget emotional information as well. In contrast to participants’ beliefs, DF effect was only found for neutral items, showing that the participants’ predictions were actually inaccurate with regards to how well they can forget emotional information.

Bailey and Chapman (2012) showed that emotional information, especially negatively emotional information can disrupt cognitive control, leading to reduced DF effects. Similarly, in the present study, the DF effect was only found for neutral items, and not for emotional ones. DF effect in item-method paradigms in which each item is followed by an instruction like in the present study is usually attributed to encoding differences between remember and forget items (Anderson & Neely, 1996; Lehman & Bovasso, 1993; MacLeod, 1999). According to this selective-rehearsal account, remember items are rehearsed more elaborately than forget items since presenting each item with a corresponding instruction creates a set differentiation. The item is kept in working memory until the instruction appears, and while a remember instruction results in further processing, a forget instruction results in the termination of processing for that item (Basden, 1996; MacLeod, 1999). This explanation has been recently challenged by an alternative account that emphasizes the role of inhibitory processes along with selective rehearsal. In this view forget instructions result in active inhibition of the items (Hauswald, Schulz, Iordanov, & Kissler, 2010). In the present study, emotionality of the items presumably disrupted their inhibition. There are also findings in the literature that do not support this explanation. Taylor, Quinlan, and Vullings (2018) investigated whether negative emotion prevents intentional forgetting and found equivalent DF effects for negative, positive, and neutral photographs and showed that individuals are capable of exerting control to intentionally forget, despite the emotional valence of the information. Additionally, Yang et al. (2012) found no difference in DF effect for neutral and negative photographs. These discrepant results require finer analyses of the types of emotional materials that are used in emotion studies and the emotional states of the participants. In one of the studies that investigated the effects of mood (i.e., positive and negative) on DF, Bäuml and Kuhbandner (2009) found that positive mood eliminated the DF effect, whereas DF effect was obtained in negative mood induction and neutral control conditions. In the present study, mood measures were collected for exploratory purposes and our results replicated previous studies showing that mood-congruent information is remembered better (Eich & Metcalfe, 1989; Payne & Corrigan, 2007) and is more resistant to intentional forgetting (Bäuml & Kuhbandner, 2009). The findings also tentatively showed that JOLs do not reflect mood congruency. Mood was not manipulated systematically in the present study. Thus, in order to reach a more definite conclusion about JOLs and mood, future studies should involve mood manipulations or systematic recruitment of participants with positive versus negative mood.

JOLs for remember items replicated previous findings showing higher retrieval predictions for emotional than neutral information (Hourihan & Bursey, 2017; Hourihan et al., 2017; Undorf et al., 2018). Although JOLs predicted better memory for negative than positive items, and better memory for positive than neutral items, actual recognition only confirmed better memory for negative item. Although, to our knowledge, there are no prior studies investigating JOLs for emotional forget items, it was hypothesized that participants would give higher JOLs for emotional items despite the forget instructions. This hypothesis was confirmed. Actual recognition was similar to remember items, with an advantage for only negative valence. Negative items were also more difficult to discriminate from the new items in the recognition test, revealed by the FA rates and recognition bias statistics.

The findings of negativity in actual recognition and heightened recognition bias for negative items are in line with studies showing enhancement of memory for negative information at the behavioral and neural level (Hauswald et al., 2010; Humphreys et al., 2010; Yang et al., 2012). Emotions influence cognitive processes by directing attention to the most relevant features of the environment (Öhman, Flykt, & Esteves, 2001; for a review, Kensinger, 2009) and by increasing arousal (Humphreys et al., 2010). Some studies found memory enhancement effects for both of positive and negative information than to neutral information (e.g., Hess et al., 2013) and others failed to find a memory advantage for positive information (e.g., Humphreys et al., 2010). Humphreys et al. reported that while no difference was observed in attentional processes for positive and negative photographs, recognition and bias were higher for negative photographs than for positive and neutral ones, possibly due to heightened arousal to negative photographs. One shortcoming of the present study is that the arousal levels for the positive and the negative items were similar, making it impossible to comment on the effects of arousal on the heightened recognition of negative items.

In the Enriched Model of Metacognition (Efklides, 2014), it is claimed that anything that can interrupt cognitive control, will lead to effortful processing, resulting in increased time to complete a task. In the present study, for exploratory purposes, RTs for providing JOLs were collected. It was found that judgments were slower for forget than remember items, when the information was emotional. Participants probably had more difficulty deciding whether they can remember emotional forget items. There may be two reasons for this. First, the participants may be sensitive to the fact that the item is emotional and that the magnitude of cognitive control they have may be different from neutral items. Emotionality made participants slower in their judgments for both positive and negative items, supporting this explanation. Possible disruptive effects of emotional information on cognitive control can explain longer response times for JOLs for emotional items. Another possibility is that judging how well a forget item will be remembered is somewhat contradictory to judge. Judgments of forgetting instead of JOLs may have been easier to provide for forget items.

Koriat, Sheffer, and Ma’ayan (2002) claim that JOLs are usually based on comparing the characteristics of the items in a list such as the nature of different materials, but not on factors such as performance differences related to memory processes. In the present study, participants underestimated their probability of recognizing items on a subsequent test. It may be the case that they were not aware of the ease of retrieval in recognition tests, in comparison to recall tests. Similarly, Carroll, Nelson, and Kirwan (1997) found that participants were underconfident in their JOLs for related paired-associates due to their lack of awareness of the effects of semantic-relatedness of the items on their memory performance. Other possible reasons for underestimation may be factors such as how much individuals trust their memory and their general self-confidence. Future studies can systematically investigate these factors.
